# Investigations of Effects of Intermetallic Compound on the Mechanical Properties and Shape Memory Effect of Ti–Au–Ta Biomaterials

**DOI:** 10.3390/ma14195810

**Published:** 2021-10-04

**Authors:** Wan-Ting Chiu, Kota Fuchiwaki, Akira Umise, Masaki Tahara, Tomonari Inamura, Hideki Hosoda

**Affiliations:** Institute of Innovative Research (IIR), Tokyo Institute of Technology, 4259 Nagatsuta-cho, Midori-ku, Yokohama 226-8503, Japan; kfuchiwaki@tenaris.com (K.F.); umise.a.aa@m.titech.ac.jp (A.U.); tahara.m.aa@m.titech.ac.jp (M.T.); inamura.t.aa@m.titech.ac.jp (T.I.); hosoda.h.aa@m.titech.ac.jp (H.H.)

**Keywords:** biomedical materials, intermetallic compound, shape memory alloys, shape memory effect, Ti–Au–Ta

## Abstract

Owing to the world population aging, biomedical materials, such as shape memory alloys (SMAs) have attracted much attention. The biocompatible Ti–Au–Ta SMAs, which also possess high X–ray contrast for the applications like guidewire utilized in surgery, were studied in this work. The alloys were successfully prepared by physical metallurgy techniques and the phase constituents, microstructures, chemical compositions, shape memory effect (SME), and superelasticity (SE) of the Ti–Au–Ta SMAs were also examined. The functionalities, such as SME, were revealed by the introduction of the third element Ta; in addition, obvious improvements of the alloy performances of the ternary Ti–Au–Ta alloys were confirmed while compared with that of the binary Ti–Au alloy. The Ti_3_Au intermetallic compound was both found crystallographically and metallographically in the Ti–4 at.% Au–30 at.% Ta alloy. The strength of the alloy was promoted by the precipitates of the Ti_3_Au intermetallic compound. The effects of the Ti_3_Au precipitates on the mechanical properties, SME, and SE were also investigated in this work. Slight shape recovery was found in the Ti–4 at.% Au–20 at.% Ta alloy during unloading of an externally applied stress.

## 1. Introduction

Shape memory alloys (SMAs) have attracted much attention in the biomedical and biomaterials communities due to their functionalities, such as shape memory effect (SME) and superelastic (SE) behavior, which could be manipulated by controlling their operation temperature and externally applied stress [1,2,3]. Since the Ni–Ti (Nitinol) alloy was utilized as SMA stent, a commercial breakthrough was made in 1990s; thereafter, SMAs have been developed and widely accepted by the public [4]. However, a high potential of Ni hypersensitivity to the human body in the Nitinol impedes its applicability [5,6]; therefore, a substitution possessing biocompatibility for the Nitinol is urgently needed. The Ti–based alloys in their β–phase have been reported to perform good SME and SE [7,8]; in addition, Ti is known as a biocompatible metal [9,10]. Therefore, biocompatible β–Ti–based SMAs were chosen in this study for the applications in biomedical materials.

For manipulation of the performance of the β–Ti alloy, gold (Au) was selected in this study for the following reasons. First, from the biological point of view, Au is also known as a metal with excellent biocompatibility [11]. Second, Au meets the essential requirement of the high X–ray contrast [12] for the applications in the medical devices, such as a guidewire for surgery. Third, from the chemical point of view, Au possesses excellent corrosion resistance in the human body fluid [13,14]. Based on the aforementioned prerequisites, Au is a potential candidate to enhance the properties of the β–Ti SMAs towards biomedical applications; therefore, the binary Ti–Au–based alloy was chosen in this work. It is necessary to mention that the near–eutectoid Ti–4 at.% Au–based alloy was designated in this study since the relatively low temperature at the eutectoid reaction facilitates the preparation of the SMAs.

Regardless of the above–mentioned promising characteristics of the binary Ti–Au–based alloy, Plichta et al. reported that the massive transformation took place during cooling in the near–eutectoid Ti–4 at.% Au alloy, resulting in the formation of the α–massive martensite [15,16]. Here, the massive transformation indicates the phase transformation of β → α_m_ (where α_m_ suggests the α–massive martensite). The α_m_ phase exhibits neither SME nor SE; thus, for revealing the functionality, such as SME and SE, a β–stabilizer, tantalum (Ta), was introduced into the near–eutectoid Ti–4 at.% Au–based alloy to stabilize the parent β–phase at operation temperature (i.e., body temperature).

Tantalum, which is known as a β–stabilizer, was chosen in this study for the manipulation of the functionalities of the Ti–Au–based SMA by tuning its martensite transformation start temperature (*M*_s_) [8]. According to the binary Ti–Ta phase diagram, regardless of the addition amount of Ta to Ti, a solid solution (i.e., isomorphous) is formed through the entire concentration range [17]. The aforementioned α–massive martensite, which performs neither SME nor SE, could be suppressed by the Ta addition and could be transformed into the functional parent β–phase and/or α′′–martensite phase [18,19,20]. Besides the purpose for revealing the functionalities, Ta, which is also a heavy element, could also contribute high X–ray contrast for the medical devices, such as guidewires [21,22]. Furthermore, concerning the biocompatibility to the human body, the Ta element, which is also considered as a promising candidate [23,24,25], shows high biocompatibility. Based on the aforementioned advantages of the Ta element, it was thus chosen as the third element for the manipulation of the Ti–Au–based SMAs in this work towards the applications of biomaterials.

For the binary Ti–Au SMAs, most of the researches focused on the near–Ti_50_Au_50_ alloys for the applications of high temperature SMAs [26,27,28]. In the case of the near–eutectoid Ti–Au alloy (i.e., Au concentration at approximately 4.2 at.% (or 15.3 wt.% Au)), the literature is relatively limited. As previously mentioned, Plichta et al. studied the α–massive martensite generated from the massive transformation around the near–eutectoid Ti–Au alloys [15,16]. Kikuchi et al. screened the elastic moduli of the Ti–Au alloys and other alloys from 5 wt.% to 30 wt.% addition amounts for the applications of biomedical materials, such as dental materials [29]. Concerning the binary Ti–Ta SMAs, Buenconsejo et al. claimed that the Ti–Ta–based alloys could be a good candidate for the applications of SMAs [30]. Ikeda et al. studied the effect of Ta addition concentration on phase constitution and the behaviors of the binary Ti–Ta alloys after aging treatment [31]. Some of the high–order Ti–Ta–based alloys have also been studied [32,33,34]. However, the ternary Ti–Au–Ta alloys, which are potential SMAs for the applications of the medical devices and biomaterials have not been investigated. This study thus investigated the unexplored effects of the Ta addition on the phase constituents, phase transformation, mechanical properties, shape memory effect, and superelasticity of the binary Ti–Au–based SMAs.

In this study, it was found that with the addition of the Ta element to the near–eutectoid Ti–Au alloy, the functionless α–massive martensite was successfully transformed into the functional β–parent phase. The intermetallic compound Ti_3_Au was formed while Ta addition amount was concentrated up to 30 at.%. Having the precipitation of the intermetallic Ti_3_Au compound, positive effect to the strength of the alloys were achieved. This work is a preliminary study of the Ti–Au–Ta–based SMAs and this ternary system is now still studying by our research group. More results concerning the Ti–Au–Ta–based alloys and their high–order systems will be published in the future.

## 2. Materials and Methods

Titanium sponges (Ti, purity = 98%), Gold plates (Au, purity > 99.9%), and Tantalum flakes (Ta, purity > 99.9%) were used as the starting materials for the preparations of the Ti–Au–Ta ingots. The purification of the Ti sponges could be found in our previous study [35]. Alloys with compositions of the Ti–4 at.% Au–20 at.% Ta and the Ti–4 at.% Au–30 at.% Ta in a total weight of eight grams were fabricated by conventional physical metallurgy techniques. In this article, these alloys are abbreviated to Ti–4Au–20Ta and Ti–4Au–30Ta, respectively, unless otherwise mentioned. The constituent elements were manufactured under an Ar–1 vol.% H_2_ atmosphere by an arc–melting system, which is equipped with a non–consumable W electrode. The ingots were re–melted seven times and were flipped upside down prior to each re–melting to affirm the chemical homogeneity of the composition in the entire ingot. The arc–melted ingots are denoted as “as–melted” specimens.

Followed by an arc–melting, the as–melted ingots were cleaned, wrapped by Ti films, vacuum sealed into quartz tubes, and homogenized at 1273 K for 7.2 ks under pure Ar atmosphere for further composition homogenization after arc–melting process. The homogenized ingots were then quenched into iced–water for the termination of the homogenization treatment and the homogenized specimens were identified by “as–HT” specimens.

The as–HT specimens were then cold–rolled into thin films at room temperature (RT; i.e., 295 ± 3 K) via the cold–rolling machine equipped with a double–deck roll. The alloys were cold–rolled until a reduction in thickness of 98% was achieved. For that alloy, which cannot be cold rolled up to reduction in thickness of 98%, process–annealing at 1273 K for 1.8 ks was conducted followed by the identical cold–rolling procedure at RT until the reduction in thickness of 98% was obtained. On the other hand, for that alloy, which cannot be cold rolled owing to its low cold workability, was hot–rolled at 1173 K until the reduction in thickness of 98%. The thinned down specimens were named as “as–rolled” alloys. Specimens with specific shapes and dimensions were cut off from the as–rolled alloys for the following analysis and measurements.

The specimens with certain shapes, which were prepared from the as–rolled alloys, were then cleaned, wrapped into Ti films, vacuum sealed into quartz tubes, and solution–treated (ST) at 1273 K for 1.8 ks under the atmosphere of pure Ar. Afterward, specimens were quenched into iced–water for the termination of the solution–treatment. The solution–treated specimens were thus named as “ST” specimens. An illustration for the aforementioned thermomechanical processes in detail could be found in our previous articles [35,36].

The ST specimens were cleaned, grinded by emery papers with different meshes, and polished by the 1 μm diamond paste to obtain fine surface finish prior to the analysis of phase identification. A *θ*–2*θ* X–ray diffraction (XRD) measurement was carried out at RT under ambient for the determination of phase constituents. Tube current and voltage were at 40 mA and 45 kV while the Cu K_α_ radiation (*λ* = 1.5405 Å) was used as the X–ray source. The scan range was from 20° to 120° with a scan rate at approximately 2°/min. A standard Si plate was used for the correction of the external system error.

Microstructure observations and chemical composition analysis were carried out by the field–emission scanning electron microscopy (FE–SEM) along with an energy dispersive X–ray spectrometry (EDS) at RT at an electron beam voltage of 30 kV. Similar to the X–ray diffraction measurements, prior to the microstructure observation and chemical composition analysis, the specimens were cleaned, mechanically grinded by emery papers, and polished by diamond paste with a diameter of 1 μm to obtain fine surface finish for the analysis.

In addition to the aforementioned fundamental characterizations, examinations of the functionalities, such as shape memory effect, were also carried out by the test of bending deformation followed by a heating process by a cigar lighter, which reached temperatures higher than 1000 K. Specimens with a dimension of 0.2 mm (thickness) × 2 mm (width) × 20 mm (width), were obtained from the above–mentioned ST specimens. The specimens were firstly subjected a 5% surface strain, which was made by bending the specimens with a round bar with a diameter of 6 mm. The surface strain made to the specimens could be calculated by following the equation of *ε* = [*h*/(2*r*)], where *ε* is surface strain, *h* is thickness of the specimen, and *r* is curvature radius, respectively. The shape recovery rates of the specimens were determined by recognizing and calculating the shape difference in the optical photos, which were taken at the (i) after bending deformation and (ii) upon heating stages. The bending tests and calculations for shape recovery rates were carried out for three times for ensuring the accuracy and also for calculations of average shape recovery rates. An image processing software, ImageJ, was used for certifying the average shape recovery rates.

For the evaluations of the mechanical properties, two different tensile tests were carried out by a universal testing machine of Shimadzu AG1kN Autograph. Specimens, which were in a dimension of 0.2 mm (thickness) × 2 mm (width) × 10 mm (width), possessed a dog–bone structure. The tensile tests were carried out at RT under ambient in two different manners: (1) the continuous tensile test and (2) the cyclic loading–unloading tensile tests. A video extensometer, which was attached to the universal testing machine, was used for the accurate measurement of shape deformation strain for both tensile tests. In the case of the continuous tensile test, the specimens were subjected to a continuous tensile stress until their fractures. On the other hand, in the case of the cyclic loading–unloading tensile tests, specimens were repeatedly subjected to a 1% strain per cycle until the 10^th^ cycle or the fractures of the specimens. All the specimens were subjected to a strain rate of 8.3 × 10−4 s^−1^ and the stress loading direction was parallel to the rolling direction (RD) of the alloys.

## 3. Results and Discussion

### 3.1. Cold Workability

In the case of the Ti–4Au–20Ta specimen, it could be cold rolled up at RT to the reduction in thickness of 70% in the first cold–rolling. Followed by the first cold–rolling, process–annealing was carried out at 1273 K for 1.8 ks. Second, cold–rolling was thereafter conducted until the reduction in thickness reached 98%. The final thickness of the specimen after cold–rolling was at approximately 0.2 mm. On the other hand, in the case of the Ti–4Au–30Ta specimen, it was found that the alloy, which showed low workability, cracked at the first pass of the cold–rolling process. Hot–rolling was therefore executed at 1173 K instead for thinning down the specimen until the reduction in thickness reached 98%. The differences in the cold workability of these two alloys are explained by the phase constituents and microstructure observations in both Section 3.2 and Section 3.3.

### 3.2. Phase Identification

**Figure 1:**
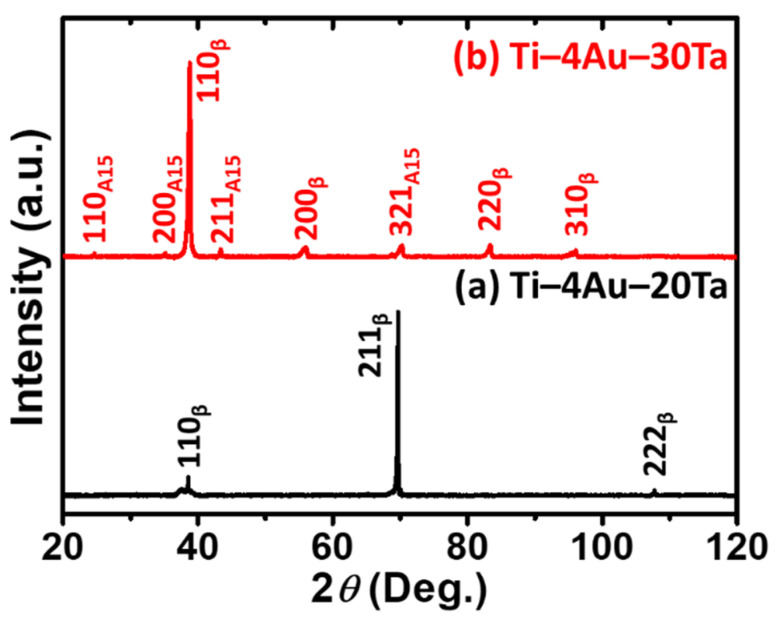
The X–ray diffraction patterns of the (**a**) Ti–4Au–20Ta alloy and the (**b**) Ti–4Au–30Ta alloy at RT under ambient.

The X–ray diffraction patterns of the (a) Ti–4Au–20Ta alloy and the (b) Ti–4Au–30Ta alloy at RT under ambient are shown in Figure 1. Put simply, the parent β–phase was found in the (a) Ti–4Au–20Ta alloy indicating a single β–phase of this alloy. On the other hand, in addition to the parent β–phase, a compound with a crystal structure of A15 was also found in the (b) Ti–4Au–30Ta alloy. According to the literature [37,38], the compound possessing A15 crystal structure corresponds to the intermetallic compound of Ti_3_Au. More analysis concerning the Ti_3_Au with an A15 crystal structure are shown in Section 3.3 via microstructure observations and chemical composition analysis.

It was reported that in the binary Ti–4Au specimen the apparent phase of the α′′–martensite or the α–massive martensite (α_m_), which were generated from the martensitic transformation or the massive transformation, were found [15,16]. Different from the binary Ti–4Au specimen, it is obvious that the parent β–phase was successfully stabilized via the introduction of the Ta element as the third element. These results observed in this study were also in a good agreement with literature [39,40].

On the other hand, according to the literature, the intermetallic compound of Ti_3_Au, which is a brittle phase and is often found in the Ti–Au–based alloys [35,38,41,42], could deteriorate the cold workability of the specimens. Therefore, in Section 3.1, the specimen of the Ti–4Au–30Ta alloy, which could not be cold–rolled at RT, could be attributed to the embrittlement brought from the Ti_3_Au precipitates. Hence, the cold workability agreed well with the phase constituents observed by the X–ray diffraction patterns (Figure 1) and the literatures [35,38,41,42]. More details of the Ti_3_Au precipitates concerning its microstructure are shown in Section 3.3.

### 3.3. Microstructure Observations and Elemental Mappings

**Figure 2:**
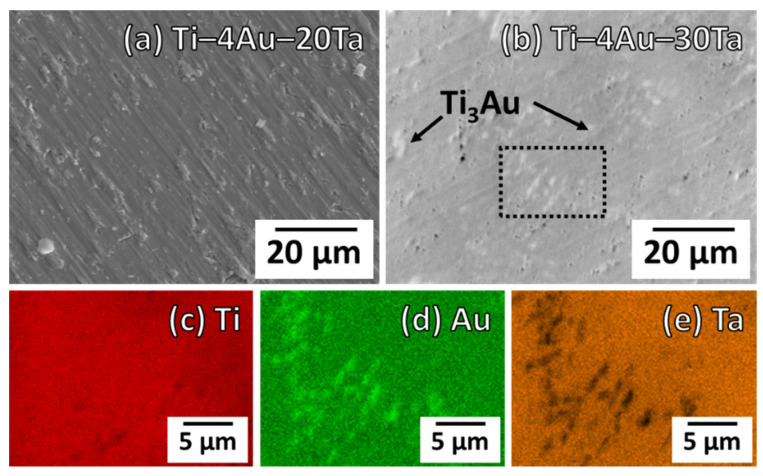
SEM images of the (**a**) Ti–4Au–20Ta alloy and the (**b**) Ti–4Au–30Ta alloy. Elemental mapping results of (**c**) Ti, (**d**) Au, and (**e**) Ta elementals of the (**b**) Ti–4Au–30Ta alloy. The elemental mapping analyzed regime of the (**b**) Ti–4Au–30Ta alloy is surrounded by a dashed square.

Figure 2a,b show the SEM images of the Ti–4Au–20Ta alloy and the Ti–4Au–30Ta alloy, respectively. In addition, the elemental mapping results of the (c) Ti, (d) Au, and (e) Ta elements in the Ti–4Au–30Ta alloy are also shown in Figure 2. The observed region of the elemental mappings is surrounded by the dashed lines in the SEM image of the Ti–4Au–30Ta alloy (Figure 2b).

In the SEM images, only single phase was observed in the Ti–4Au–20Ta alloy (Figure 2a) and this result is in accordance with its X–ray diffraction pattern (Figure 1a), where merely the β–parent phase was found. On the other hand, it is clear that some precipitates in bright contrast were discerned in the Ti–4Au–30Ta alloy (Figure 2b). The size of the oval–shaped precipitates was approximately 1–3 μm and the volume fraction of the precipitates was about 5%. It is also worth mentioning that the microstructure of the precipitates was found very similar with those in the literature [38]. More details concerning the precipitates in the Ti–4Au–30Ta alloy are explained by the elemental mapping results in the following.

The elemental mapping results of the precipitates were further carried out and are shown in Figure 2c–e. Bright contrast suggests high concentration while dark contrast indicates low concentration. It was found that, in the case of Ti element, there was no obvious concentration difference between the matrix and the precipitates (Figure 2c). On the other hand, the precipitates were high in Au (Figure 2d) and low in Ta (Figure 2e). Given that the Ti_3_Au phase was determined by the X–ray diffraction pattern of the Ti–4Au–30Ta alloy (Figure 1b) and the literature [35,38,41,42], it thus could be concluded that the precipitates in bright contrast could be corresponded to the Ti_3_Au phase. In addition, it was reported that the Ti_3_Au phase is a line compound [37]; therefore, the solubility of Ta was extremely low (i.e., dark contrast) to the Ti_3_Au intermetallic compound. A good agreement was reached among the X–ray diffraction observations (Figure 1), SEM images (Figure 2b), elemental mapping results (Figure 2c–e), and also the literature [35,37,38,41,42].

Based on the aforementioned results, the addition of Ta as the third element promoted the precipitation of Ti_3_Au intermetallic compound since the overall chemical composition in terms of the binary Ti–Au system shifted to the Au–rich side [37] while Ta was introduced into the binary Ti–Au system. Therefore, obvious precipitates of the oval–shaped Ti_3_Au intermetallic compound with a bright contrast were observed in the Ti–4Au–30Ta alloy (Figure 1b–e).

### 3.4. Bending Test

**Figure 3:**
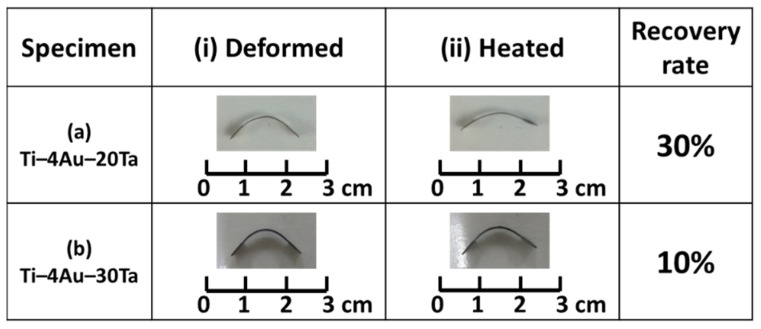
Bending tests of the (**a**) Ti–4Au–20Ta alloy and the (**b**) Ti–4Au–30Ta alloy for the examinations of the shape memory effect and shape recovery rates. Stage (**i**) corresponds to after bending deformation while stage (**ii**) indicates shape recovery upon heating process.

Results of the bending tests are shown in Figure 3. In Figure 3, column (i) indicates the specimens after bending deformation (stage i) while column (ii) suggests the shape recovery of the specimens upon heating (stage ii). Judging from the optical images, an obvious shape recovery was found in the Ti–4Au–20Ta alloy (Figure 3a); on the contrary, the shape recovery rate of the Ti–4Au–30Ta alloy (Figure 3b) was relatively faint. The shape recovery rates of (a) the Ti–4Au–20Ta alloy and (b) the Ti–4Au–30Ta alloy were determined to be 10% and 30%, respectively. Since the apparent phase of the (a) Ti–4Au–20Ta alloy was determined to be the single parent β–phase via the X–ray diffraction observation (Figure 1a) and the microstructure observation (Figure 2a), it thus could be deduced that the shape recovery behavior of the Ti–4Au–20Ta alloy was brought by the stress–induced martensite transformation (SIMT) during bending deformation (stage i) and followed by its backward transformation upon heating (stage ii).

Similar to the (a) Ti–4Au–20Ta alloy, the apparent phase of the (b) Ti–4Au–30Ta alloy was also the parent β–phase before deformation; however, precipitates of Ti_3_Au compound was also found in the Ti–4Au–30Ta alloy (Figure 1b and Figure 2b–e). The deteriorated shape recovery rate of the (b) Ti–4Au–30Ta alloy could be attributed to the suppression of the phase transformation by the Ti_3_Au precipitates during bending deformation and shape recovery. These results are in accordance with those in the literature [38]. In addition to the inhibition of the phase transformation by the Ti_3_Au precipitates, the relatively low shape recovery rates (i.e., 10% and 30%) of these two alloys could also be ascribed to the plastic deformation, which was introduced into the alloys during bending deformation.

### 3.5. Continuous Tensile Tests

**Figure 4:**
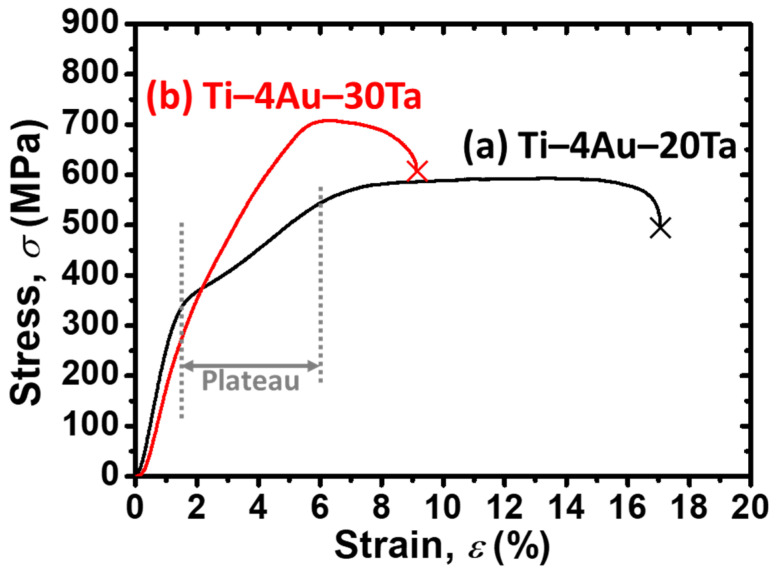
Stress–strain (SS) curves of the (**a**) Ti–4Au–20Ta alloy and the (**b**) Ti–4Au–30Ta alloy via the continuous tensile tests. The cross symbols at the end of the curves suggest fractures of the specimens. A stress plateau in the (**a**) Ti–4Au–20Ta alloy was indicated by the vertical dashed lines and the horizontal solid arrows.

Stress–strain (SS; *σ*–*ε*) curves of the (a) Ti–4Au–20Ta alloy and the (b) Ti–4Au–30Ta alloy by the continuous tensile tests are shown in Figure 4. The cross symbols at the end of the SS curves suggest the fractures of the specimens. The strain until fracture of the (a) Ti–4Au–20Ta alloy was at approximately 17%, which was almost twice larger than that of the (b) Ti–4Au–30Ta alloy (i.e., deformation strain at approximately 9%), showed high elongation. The different ductility of these two alloys could be elaborated through the following reasons.

Firstly, it was worth noticing that in the (a) Ti–4Au–20Ta alloy, there is a clear stress plateau, which is indicated by the vertical dashed lines and the solid horizontal line with arrows. Given that the apparent phase of the (a) Ti–4Au–20Ta alloy is the parent β–phase at the testing temperature (i.e., RT) (Figure 1a), the stress plateau could be attributed to the stress–induced martensitic transformation (SIMT) [38,41,43]. The first yielding could be attributed to the onset of the SIMT, while the second yielding could be ascribed to the commencement of the plastic deformation [38,41,43]. On the other hand, almost no stress plateau was found in the case of the (b) Ti–4Au–30Ta alloy. Therefore, the relatively high elongation of the (a) Ti–4Au–20Ta alloy could be ascribed to the SIMT. It is also worth mentioning that the SIMT found in the Ti–4Au–20Ta alloy in Figure 4a corresponds well with the result of the bending test in Section 3.4. While the Ti–4Au–30Ta alloy, which had a lower shape recovery rate upon heating (Figure 3b), showed almost no stress plateau in Figure 4b.

Secondly, the Ti_3_Au intermetallic compound, which was merely found in the (b) Ti–4Au–30Ta alloy (Figure 1b and Figure 2b–e), is known as a brittle phase [44]. The ductility of the (b) Ti–4Au–30Ta alloy was thus deteriorated by the formation of the Ti_3_Au precipitates. On the contrary, the (a) Ti–4Au–20Ta alloy showed relatively high deformation strain before fracture since no brittle Ti_3_Au precipitates were found in this alloy (Figure 1a and Figure 2a).

To sum up, the relatively high elongation of the (a) Ti–4Au–20Ta alloy could be attributed to the occurrence of SIMT and lack of Ti_3_Au precipitates. On the other hand, the relatively low elongation of the (b) Ti–4Au–30Ta alloy was due to the Ti_3_Au precipitates and almost free of SIMT during deformation.

However, reverse to the ductility, comparatively high strength was observed in the (b) Ti–4Au–30Ta alloy than that of the (a) Ti–4Au–20Ta alloy in the continuous tensile tests (Figure 4). It was found that the ultimate tensile strength (UTS) was at approximately 737 MPa for the (b) Ti–4Au–30Ta alloy and at approximately 600 MPa for the (a) Ti–4Au–20Ta alloy. This could be simply explained by the precipitation–hardening effect brought from the precipitates of the Ti_3_Au compound and also the solution–hardening effect brought by the Ta addition as the third element.

### 3.6. Functional Mapping

**Figure 5:**
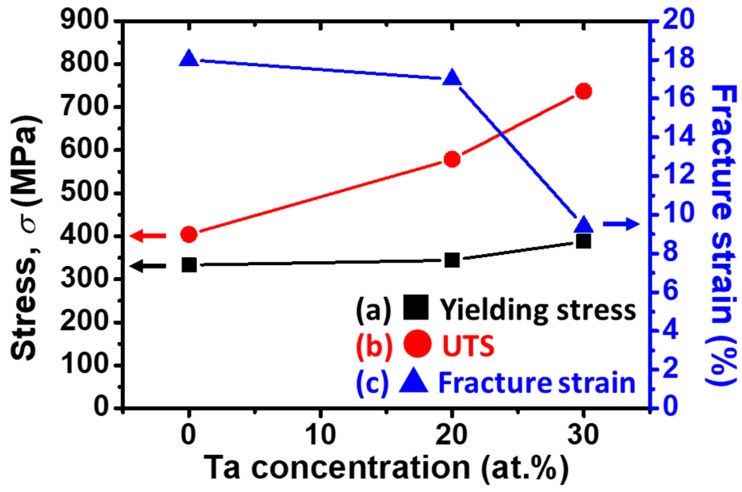
Functional mapping of (**a**) yielding stress (black squares), (**b**) ultimate tensile strength (UTS) (red circles), and (**c**) fracture strain (blue triangles) as a function of the Ta addition concentration in the alloys. (Left *y*–axis: stress (MPa); Right *y*–axis: fracture strain (%)) (Note that the results of the binary Ti–4Au alloy was cited from our preliminary research [35]).

A functional mapping, which reveals (a) yielding stress (black squares), (b) ultimate tensile strength (UTS) (red circles), and (c) fracture strain (blue triangles) as a function of Ta concentration in the alloys, is shown as Figure 5. It is necessary to mention that for the purposes of completeness and comparison, the binary Ti–4Au alloy was cited from our preliminary research [35] and plotted in Figure 5. It is obvious that both the (a) yielding stress and the (b) UTS increased monotonically with Ta addition concentration. Similar to the explanation in Section 3.5, this enhancement in strength could be attributed to the solid–solution strengthening effect of the introduction of Ta element and the precipitation hardening effect caused by the Ti_3_Au precipitates. Slip of dislocation (i.e., plastic deformation) was inhibited by the introduction of the Ta impurity and the Ti_3_Au precipitates. Opposite to the strength, fracture strain (Figure 5c) of the specimens decreased with the introduction of Ta concentration due to the precipitation of the brittle Ti_3_Au intermetallic compound.

### 3.7. Cyclic Loading–Unloading Tensile Tests

**Figure 6:**
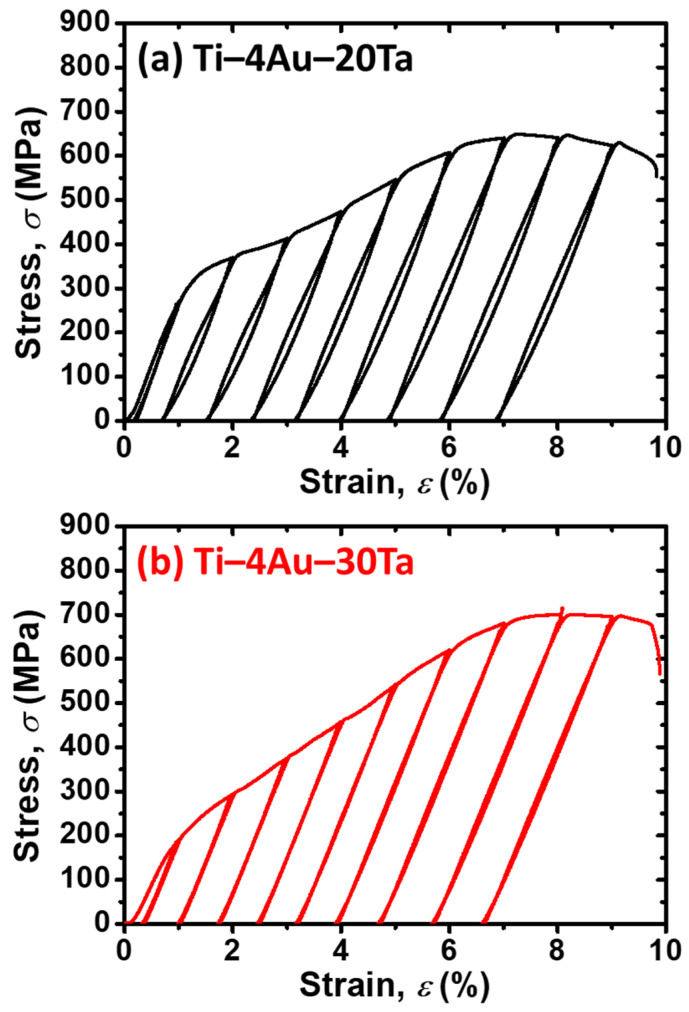
Cyclic loading–unloading tensile tests of the (**a**) Ti–4Au–20Ta alloy and the (**b**) Ti–4Au–30Ta alloy at RT under ambient.

The cyclic loading–unloading tests of the (a) Ti–4Au–20Ta alloy and the (b) Ti–4Au–30Ta alloy are shown in Figure 6. Overall, it was found that the (a) Ti–4Au–20Ta alloy which exhibited slight pseudoelasticity (PE), showed slight shape recovery during unloading. While the (b) Ti–4Au–30Ta alloy showed almost no shape recovery during unloading in the cyclic loading–unloading tests at RT. Therefore, it could be deduced that the austenite transformation start temperature (*A*_s_) is higher than the operation temperature (i.e., RT). In addition, the vanished shape recovery in the (b) Ti–4Au–30Ta alloy could be ascribed to the inhibition of the Ti_3_Au precipitates. On the other hand, the slight shape recovery in the (a) Ti–4Au–20Ta alloy could be attributed to the pseudoelasticity via the reorientation of martensitic variants, which is commonly observed in the martensite phase [45,46,47]. Moreover, similar to the results from the continuous tensile tests (Figure 4) in Section 3.5, the (b) Ti–4Au–30Ta alloy showed improved strength than that of the (a) Ti–4Au–20Ta alloy.

**Figure 7:**
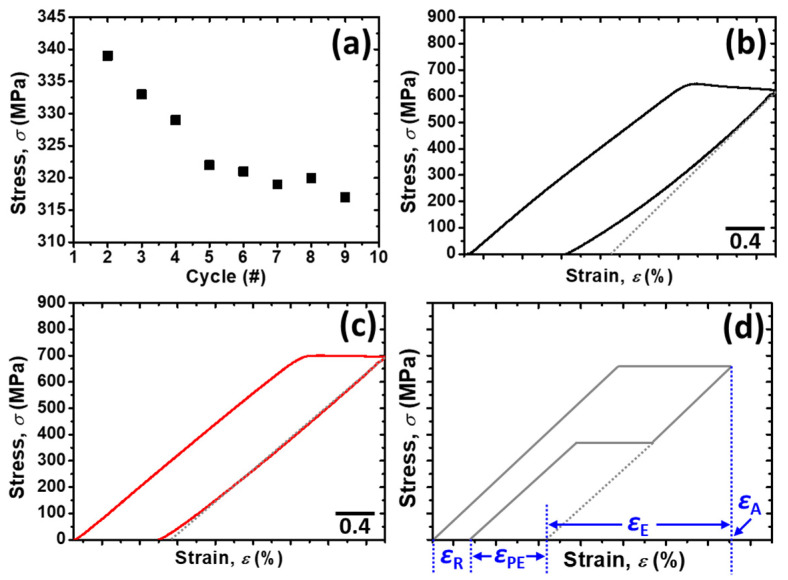
(**a**) Stress for the first yielding stress (i.e., stress for SIMT) of the Ti–4Au–20Ta alloy, (**b**) the ninth cycle in the cyclic loading–unloading tensile test of the Ti–4Au–20Ta alloy, (**c**) the ninth cycle in the cyclic loading–unloading tensile test of the Ti–4Au–30Ta alloy, and (**d**) the illustration for the explanations of the terms in the SS curve. Where *ε*_A_ indicates the total applied strain, *ε*_E_ suggests the elastic shape recovery strain, *ε*_PE_ corresponds to shape recovery strain brought by pseudoelasticity, and *ε*_R_ represents the remaining strain after unloading.

For further analysis of the pseudoelasticity, every cycle in the Ti–4Au–20Ta alloy was detached and the stress for SIMT (i.e., first–yielding stress), which is shown in Figure 7a, was read from every cycle of the cyclic loading–unloading tensile test (Figure 6a). During the cyclic tensile test, dislocations, which increased the internal stress, was introduced into the specimen. The martensite phase was stabilized by the elevated internal stress; as a result, the external stress for the SIMT (*σ*_SIMT_) was reduced accordingly (Figure 7a). This phenomenon was in good agreement with that in the literature [41].

The ninth cycle in the cyclic loading–unloading tensile test of the Ti–4Au–20Ta specimen is also shown in Figure 7b. It is obvious that during unloading of externally applied stress, there was a slight shape recovery, which is at approximately 0.5%, was brought from the pseudoelasticity. The shape recovery originated from the pseudoelasticity was determined by reading strain difference between the overall recovery strain after fully unloading and the strain produced by the elastic shape recovery. Further explanations for the specific terms are shown and explained by the illustration in Figure 7d.

On the other hand, almost no shape recovery was found in the Ti–4Au–30Ta alloy (Figure 7c) during unloading of the externally applied stress. The aforementioned Ti–4Au–20Ta alloy and Ti–4Au–30Ta alloy results are in good accordance with those in the bending tests (Figure 3) and the continuous tensile tests (Figure 4). An illustration for the explanations of the specific terms are shown in Figure 7d. In Figure 7d, *ε*_A_ indicates the total applied strain, *ε*_E_ suggests the elastic shape recovery strain, *ε*_PE_ corresponds to shape recovery strain brought by pseudoelasticity, and *ε*_R_ represents the remaining strain after unloading.

This study worked on the Ti–4Au–20Ta alloy and Ti–4Au–30Ta alloy for the preliminary screening of the Ti–Au–Ta–based shape memory alloys. Some of the Ti–Au–based studies have already been carried out by our research group [35,36,38,41,43] while some of the Ti–Au–based studies are now under investigations and preparations. Further results concerning the Ti–Au–based shape memory alloys will be published in the future by our research group.

## 4. Conclusions

For the biomedical applications in this work, the Ti–4Au–20Ta and the Ti–4Au–30Ta alloys were prepared by physical metallurgy techniques and their phase constituents, composition analysis, mechanical behaviors, and functional properties, such as shape memory effect (SME) and pseudoelasticity (PE), have been studied. The important findings are listed in the following points.

1. The single parent β–phase was found in the Ti–4Au–20Ta alloy while the parent β–phase along with the precipitates of the Ti_3_Au intermetallic compound in a volume fraction of approximate 5% was observed in the Ti–4Au–30Ta alloy.
2. The Ti–4Au–20Ta alloy showed about 30% shape recovery upon heating in the bending tests while only 10% shape recovery was found in the Ti–4Au–30Ta alloy due to the inhibition of phase transformation between austenite and martensite from the Ti_3_Au precipitates.
3. An obvious two–stage yielding, which corresponds to the stress for the stress–induced martensite transformation (SIMT) and the stress for plastic deformation, was found in the Ti–4Au–20Ta alloy. On the other hand, no obvious two–stage yielding was found in the Ti–4Au–30Ta alloy due to the inhibition of SIMT from the Ti_3_Au intermetallic compound.
4. In the continuous tensile test, the strength of the Ti–4Au–30Ta alloy was higher than that of the Ti–4Au–20Ta alloy due to the truth of the solid–solution hardening effect from the Ta addition as the third element and the precipitation hardening effect from the Ti_3_Au intermetallic compound. On the contrary, better elongation was found in the Ti–4Au–20Ta alloy than that of the Ti–4Au–30Ta alloy owing to the SIMT and no precipitation of Ti_3_Au compound.
5. Slight pseudoelasticity at approximately 0.5% was found in the Ti–4Au–20Ta alloy showing slight shape recovery after unloading of externally applied stress while almost no shape recovery was found in the Ti–4Au–30Ta alloy due to the inhibition of Ti_3_Au precipitates. These results are in accordance with the bending tests and the continuous tensile tests.

## Data Availability

All data contained within the article.
